# Determination of dosage compensation of the mammalian X chromosome by RNA-seq is dependent on analytical approach

**DOI:** 10.1186/1471-2164-14-150

**Published:** 2013-03-06

**Authors:** Nathaniel K Jue, Michael B Murphy, Seth D Kasowitz, Sohaib M Qureshi, Craig J Obergfell, Sahar Elsisi, Robert J Foley, Rachel J O’Neill, Michael J O’Neill

**Affiliations:** 1 Department of Molecular and Cell Biology, University of Connecticut, 354 Mansfield Rd. U-2131, Storrs, CT 06235, USA

**Keywords:** RNA-seq, X chromosome, Dosage compensation

## Abstract

**Background:**

An enduring question surrounding sex chromosome evolution is whether effective hemizygosity in the heterogametic sex leads inevitably to dosage compensation of sex-linked genes, and whether this compensation has been observed in a variety of organisms. Incongruence in the conclusions reached in some recent reports has been attributed to different high-throughput approaches to transcriptome analysis. However, recent reports each utilizing RNA-seq to gauge X-linked gene expression relative to autosomal gene expression also arrived at diametrically opposed conclusions regarding X chromosome dosage compensation in mammals.

**Results:**

Here we analyze RNA-seq data from X-monosomic female human and mouse tissues, which are uncomplicated by genes that escape X-inactivation, as well as published RNA-seq data to describe relative X expression (RXE). We find that the determination of RXE is highly dependent upon a variety of computational, statistical and biological assumptions underlying RNA-seq analysis. Parameters implemented in short-read mapping programs, choice of reference genome annotation, expression data distribution, tissue source for RNA and RNA-seq library construction method have profound effects on comparing expression levels across chromosomes.

**Conclusions:**

Our analysis shows that the high number of paralogous gene families on the mammalian X chromosome relative to autosomes contributes to the ambiguity in RXE calculations, RNA-seq analysis that takes into account that single- and multi-copy genes are compensated differently supports the conclusion that, in many somatic tissues, the mammalian X is up-regulated compared to the autosomes.

## Background

Chromosome-based sex determination systems are most often characterized by heterotypic sex chromosomes, with one sex carrying at least one degenerate homolog [1-3]. Heterokaryotypy may result from differential gene loss or gain as the sex chromosome complement evolves from an ancestral homologous pair. Depending on the extent of the loss or gain, and the dosage sensitivity of genes on the incipient sex chromosomes, natural selection may favor the evolution of compensating mechanisms to balance expression between the sexes and between the sex chromosomes and autosomes. This can be accomplished either by up-regulating expression of sex-linked genes in the heterogametic sex or by down-regulating expression in the homogametic sex in relation to the autosomes. In *Drosophila*[4] and *Sciara*[5], genes on the single X chromosome in males are transcriptionally up-regulated, while in the nematode worm, *Caenorhabditis elegans*, the two X chromosomes in hermaphrodites are down-regulated to equal that of the XO males [6]. In contrast, for organisms displaying female heterogamety, such as birds, evidence of sex chromosome dosage compensation is lacking [7-10]. The differences in compensating mechanisms, or lack thereof, will likely reflect the relative content of haplosufficient vs. haploinsufficient genes on the sex chromosomes, but will also reflect early events of sex chromosome evolution, outcomes of sexual selection and sexual conflict, and the life history of the organism [11].

In eutherian mammals and marsupials, sex chromosome dosage compensation is achieved by global inactivation of one of the two X chromosomes in females. X chromosome inactivation (XCI) in eutherians is initiated by the expression of the *XIST* non-coding RNA just prior to implantation of the embryo, leading to heterochromatinization of one of either parental X chromosome in the fetus [12]. X-inactivation in marsupials also involves heterochromatinization of one X, governed by a non-coding RNA, *RSX*, with *XIST*-like properties, but the paternal X is exclusively chosen for inactivation [13,14].

Halving the apparent dosage of X-linked genes in female mammals via XCI presents an evolutionary conundrum: if sex chromosomes evolve from an ancestral autosomal pair, it is the heterogametic sex that would be impelled to compensate for the complete loss or degradation of the evolving Y. In other words, since female mammals never receive a Y chromosome, it is difficult to see how loss of gene dosage from the evolving Y would have any influence on regulation of X genes in females. The simplest compensating step in response to attritional gene loss from the incipient Y would be *cis*-regulatory change or *cis*-gene duplication, i.e. genetic mutation, of genes on the X. In *Drosophila*, a male-specific epigenetic mechanism of dosage compensation spares the homogametic female a potentially detrimental up-regulation of X-linked genes. If, however, compensation is achieved by genetic mutation, selection would favor epigenetic down regulation in females. Ohno recognized this and hypothesized that down-regulation of X-linked genes might evolve in response to regulatory changes to the X that are transmitted from father to daughter [15]. This would appear to be the scenario played out in *C. elegans* and mammals. Regardless of the eventual dosage compensation mechanism settled upon, the first step in compensating for gradual haploinsufficient gene loss on the Y must be an increase in transcription of surviving genes on the X in males.

Ohno’s hypothesis appeared to be borne out in three recent reports [16-18], which each showed by microarray-based transcriptome analysis that the single active X chromosome in both males and females in several eutherian species was expressed at or near a 1:1 ratio to the averaged expression of the diploid autosomal complement, termed the “X:A ratio”. However, this work was called into question by He and colleagues [19] who, through analysis of high throughput transcriptome sequence (RNA-seq) data from various tissues from human and mouse, concluded that the X:A ratio of gene expression was closer to 0.5, indicative of a lack of X-linked gene up-regulation. Xiong *et al*. report that the former studies were compromised by apparent compression of expression differences; a factor they argue is inherent to microarray expression analysis. Recently, Disteche and colleagues published a report re-analyzing RNA-seq data from [19] as well as new RNA-seq data from human cells and tissues and arrive at the conclusion that the mammalian X chromosome is upregulated in relation to autosomes [20]. Additionally, three other studies [21-23] following on the heels of [20] also report up-regulation of the mammalian X. However, in reply to these reports, He and colleagues maintain their conclusion that Ohno’s hypothesis is “invalid” [24].

The widely divergent conclusions, i.e. compensation vs. no compensation, of these studies highlight the dramatic differences in biological conclusions that can be drawn from different analytical approaches applied to similar or even identical next-generation sequence datasets. As the recent controversy over RNA editing illustrates [25-28], even though the computational tools available for next-generation sequencing analysis may be vetted in the literature, parameters that can profoundly affect outputs are often applied haphazardly. In this report we consider several issues that may contribute to variation in calculating the whole chromosome expression values that form the basis of conclusions drawn regarding the relative expression of X-linked genes to that of autosomal genes. We compared the global transcriptional output of the X chromosome with that of the autosomes using our own RNA-seq datasets and those utilized in [19] and [20] that are publically available [29-31]. Our analysis also includes RNA-seq data we have generated from X monosomic mouse and human tissues. Since X monosomy obviates X-inactivation, results from X monosomic samples are not confounded by the effects of X-linked genes that escape inactivation. We report that assumptions made in dataset trimming and several factors integral to the implementation of RNA-seq quantitative analysis have a large effect on the global calculation of the relative expression of the X chromosome to that of autosomes.

## Results

### Data distribution and treatment of outliers

Unless otherwise stated, gene expression levels are represented as FPKM (fragments per kilobase of exon per million fragments mapped). In their recent study reporting mammalian X chromosome to autosome expression ratios (X:A) equal to ~0.5, He and colleagues utilized RNA-seq datasets that then were truncated by removing substantial proportions of both highly and lowly expressed loci in order to exclude the effect of FPKM values at or near 0 while arbitrarily preserving a median value for statistical testing [19]. Contrarily, Disteche and colleagues contend that compensation of the mammalian X, can only be discerned once the skewed content toward reproductive genes on the X is taken into account [20]. Nevertheless, they too only detect compensation in most human tissues once genes with FPKM ≤ 1 are excluded. Likewise, each of the other reports addressing the Ohno controversy [21-23] disregard genes with FPKM ≤ 1, or as in [24] RPKM < 3. However, FPKM determination is not absolute and can vary significantly based on sequencing depth, sequencing platform, RNA source and other factors [32]. Moreover, since functional genes expressed at any level may be subject to selection for dosage compensation, exclusion of data based on the level of expression may skew final analysis.

**Figure 1:**
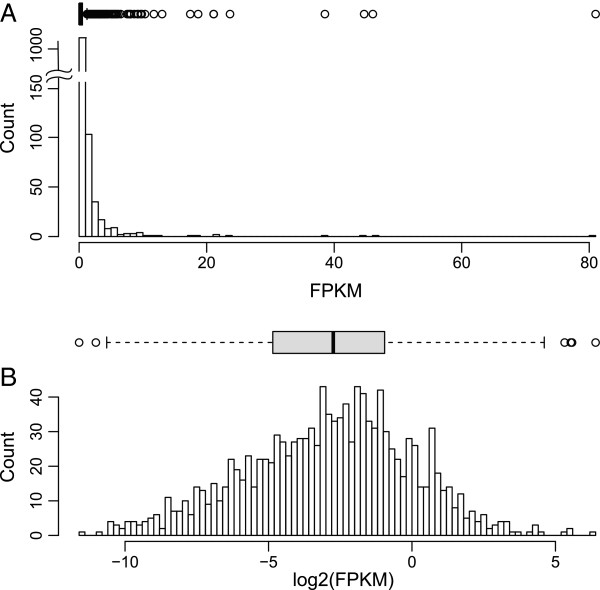
**Box plots and frequency histograms reveals log**_**2**_**-transformed FPKM values following a “normal” distribution.** (**A**) Untransformed and (**B**) log_2_-transformed FPKM values, for chromosome 1 from the human lymphoblast 45,X^m^ sample.

In our analysis, the distribution of raw FPKM values, calculated using Cufflinks v1.0.3 (see below), showed a marked shift towards lowly expressed genes (example shown in Figure 1A), and the clustering of raw FPKM values near 0 created a large number of identified outliers in the dataset. Log_2−_transformation of all FPKM values allows datasets to be more normally distributed, lending greater accuracy to summary statistics while drastically reducing the number of outliers (Figure 1B). Data was only filtered out of these analyses if it was identified as a statistical “outlier” as determined by evaluating the properties of the distribution of the data rather than by arbitrarily designated thresholds (Additional file 1: Table S1). Using this method the X:A ratio is replaced by computing the differences in the mean chromosome-wide estimates of log-transformed FPKM values (i.e. relative X expression (RXE) = log_2_(X) – log_2_(A)). Because of the shift to a log_2_-scale, if the X and autosomal expression means are equal, then the difference between those values will be 0, indicating dosage compensation is occurring. Any positive value represents higher X chromosome expression and negative values represent, on average, higher autosomal gene expression. A lack of dosage compensation between the X and autosomes would yield a value equal to −1 (a two-fold higher level of expression for the autosomes or an X:A ratio of 0.5).

### Mapping parameters in measuring chromosome-wide gene expression

We found that altering the parameters used by various software packages to map RNA-seq data to reference genomes has a profound impact on the calculation of chromosome-wide expression values. To highlight shifts in global expression estimates created by solely employing different mapping parameters, we calculated RXE with three different parameter sets implemented in Bowtie v0.12.7. In their recent RNA-seq study of X:A ratios, Xiong and colleagues [19] implemented what has been termed “unique” mapping parameters to map their datasets, meaning each short sequence read is aligned to the best position in the genome while any read which maps to multiple positions is excluded from the output. Mapping all 10 of our datasets using unique parameters yielded lower RXE values than other mapping approaches (Table 1). Estimates of RXE for a variety of tissues range from −1.43 to −0.32 (or an X:A ratio of 0.37 and 0.8, respectively), describing a level of general X-expression less than that of autosomal expression and well within the range of RXE that would characterize a system with no dosage compensation.

**Table 1:** RXE based on mapping parameters

		**Mapping parameter**	
**Tissue**	**Unique**	**Non-unique**	**Non-unique splicing**
X^m^, Human lymphoblast	−0.86	−0.34	0.21
X^p^, Human lymphoblast	−0.92	−0.54	−0.04
Human lymphoblast	−0.75	−0.42	−0.20
Human brain	−0.32	−0.15	−0.00
Human liver	−1.43	−1.06	−0.79
Mouse brain	−0.68	−0.53	−0.20
X^m^, mouse brain	−0.99	0.88	−0.70
X^p^, mouse brain	−0.76	−0.88	−0.96
XX, Mouse Brain	−0.87	−0.82	−0.86
XY, mouse brain	−0.45	−0.44	−0.60

Relative X-chromosome expression values based on mapping parameters. Numbers based on calculations from raw number of reads mapped. No annotation was used to delineate between genetic categorical groups (i.e. exon, introns, etc.). All ratios were log_2_-transformed to maintain consistency with other expression values.

Ostensibly, unique mapping parameters are employed to create FPKM values while avoiding potential confounding effects of including genes that are erroneously counted as “expressed” due to cross-mapping of short reads to multiple loci. However, paralogous gene families having even short segments of high similarity will be completely excluded by such methods. Since gene duplication is one potential means of achieving dosage compensation upon loss of a homolog, we examined the relative X chromosome content for highly similar paralogous gene families (> 70% sequence similarity) compared to the autosomes. For human we found ~2 fold enrichment for paralogous gene families on the non-recombining portion of the X chromosome compared to autosomes, and ~1.5 fold enrichment for mouse (Additional file 2: Table S2). Such enrichment means that when only unique mapping parameters are considered, the X chromosome would be more likely to have reads excluded as compared to autosomes, skewing the estimates of RXE downward. To more accurately account for paralogous transcripts/genes, we implemented a mapping approach, termed “non-unique”, that aligns each read only to the best fit position in the genome but does not exclude reads that map to multiple positions.

**Figure 2:**
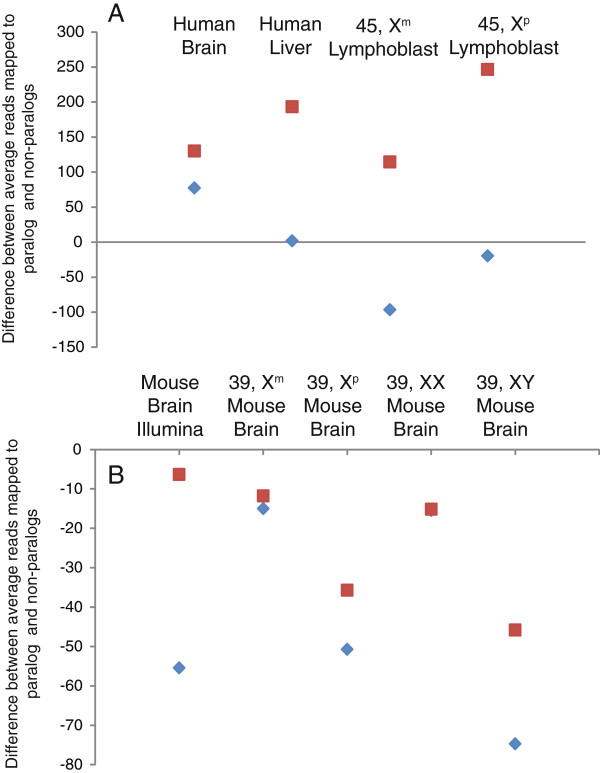
**The mapping algorithm differences between the average (per gene) number of reads mapped to paralogs (>70% similarity) vs. average (per gene) number of reads mapped to non-paralogs.** All human (**A**) and most mouse (**B**) tissues show a disproportionate effect of mapping to paralogs. Y-axis values indicate the difference in the number of reads mapped to paralogs averaged across all paralogs as compared to the number of reads mapped to non-paralogs averaged across all non-paralogs. Blue diamonds indicate the usage of a “unique” mapping approach. Red squares indicate the usage of a “non-unique” mapping approach.

Mapping with non-unique parameters in Bowtie yielded RXE estimates ranging −1.06 to −0.15, describing both a lack of dosage compensation (RXE to be at half that of A in some cases) and dosage compensation (RXE = 0, i.e. X and A almost equal) (Table 1). RXE for most tissues increased (values of the index moved close to zero) as compared to unique mapping runs. We also found a consistent increase in the number of reads mapped to paralogs in non-unique mapping runs versus unique mapping runs for both mouse and human tissues (Figure 2). The smallest libraries in the analysis, normal mouse XX brain and 39, X^m^ mouse brain, showed the least amount of change, consistent with the notion that the ability to map paralogs is highly dependent upon sequencing depth (see below).

The report of Xiong and colleagues ignored alternative splicing in mapping program implementation, as reads spanning splice junctions were discarded. Mapping our datasets using TopHat, which considers both paralogous transcripts and splice site junctions (referred to hereafter as “non-unique, spliced” mapping), shifted RXE levels to a range of −0.96 to 0.21 (Table 1, Additional file 3: Table S3, Additional file 4: Table S4) [33,34]. Consideration of splicing pushed estimates of RXE both up or down, depending on the library examined; however, many of the estimates for a specific tissue increased their estimates of RXE. Also, three of the 10 datasets showed a twofold or greater up-regulation of the X chromosome.

For a deeper understanding of how paralogs may be affected by mapping protocol we compared RXE of non-paralogous genes, all paralogs, and *cis-* versus *trans-*paralogs (“*cis*” meaning paralogs that have duplicated on the same chromosome; “*trans*” meaning paralogs that have translocated or duplicated across multiple chromosomes). Results of RXE analyses show that, in general, paralogs are more likely to be involved in dosage compensation than non-paralogs (Table 2). Across all five tissue-specific datasets, incorporating two different methods of library construction, RXE values for all paralogs were higher than all non-paralogous genes. Moreover, across these same tissues, RXE values for *cis-*paralogs were higher than for *trans*-paralogs. We also found a considerable effect for overall activity of the X within a tissue. For instance, in brain where 34.3% of X genes are identified as active, RXE was 0.16, while in liver where the X is relatively less active (30.5% of genes active), RXE is −0.28. While this pattern holds in all comparisons, it is most pronounced when considering *cis-*paralogs.

**Table 2:** RXE across paralogs

	**Human brain**	**Human liver**	**Human lymphoblast 45, X**^**m**^	**Human lymphoblast 45, X**^**p**^	**Human lymphoblast**
cis-paralogs	**2.57 (41, 30)**^**†**^	0.18 (31, 28)	0.51 (13, 18)	0.79 (14, 17)	0.24 (38, 31)
trans-paralogs	−0.64 (76, 43)^*^	0 (69, 40)	0.20 (56, 31)	−0.55 (61, 63)	−0.06 (77, 44)
all paralogs	**0.49 (106, 65)**^**†**^	0.03 (93, 60)	0.14 (66, 43)	−0.15 (68, 46)	0.19 (104, 70)
non-paralogs	−0.18 (415, 546)^*^	−0.5 (378, 520)^*^	−0.32 (269, 393)^*^	−0.38 (272, 412)^*^	−0.73 (400, 559)^*^

Relative X-chromosome expression values across both *cis* and *trans*-paralogs for five human libraries. *Cis*-paralogs show an increased RXE compared to *trans*-paralogs and non-paralogous elements. Bolded value identifies case where X chromosome and autosome expression are significantly different from each other (p < 0.05, Kolmogorov-Smirnov test, with bootstrapping −1000 replicates). Cross indicates X chromosome expression greater than autosomes, while asterisk indicates X chromosome expression less than autosomes.

### Genome annotation

In the analysis of RNA-seq data it is customary to use one of several available reference genome annotations when mapping sequence reads and calculating FPKM. Mapping short sequence reads to a reference genome removes sequences/transcripts that arise from experimental or transcriptional noise. In calculating RXE, we utilized six different annotations: RefSeq; RefSeq (protein coding); Ensembl (gene); Ensembl (transcript); UCSC (hg19) known genes; and Gencode. These annotations were each implemented on four “non-unique, spliced” mapping files from the following datasets: human liver, human brain, 45, X^m^ and 45, X^p^ human lymphoblastoid cell lines (Table 3). RefSeq generally consists of genetic annotations that are non-redundant and supported by explicit relationships between nucleotide and protein sequences. Gencode contains these same types of annotations, but also uses computational methods to predict other genes that are then validated manually. These other structures result in the addition of more alternatively transcribed variants, non-coding loci, and pseudogenes to this annotation as opposed to RefSeq, but evidence requirements for inclusion may be lower in some cases. The Ensembl annotation incorporates additional computational steps, outside database resources, and evidence testing, which further add additional structures to the reference annotation. The UCSC annotation is generally less conservative than RefSeq and includes gene predictions for both protein-coding and non-coding RNA genes. It is clear from our analysis that estimates of RXE can vary dramatically, even within a tissue type, depending on which annotation is implemented in the transcript assembly step (i.e. within Cufflinks) (Table 3). For instance, the 45, X^m^ lymphoblastoid cell line dataset showed the greatest variability between annotations with the Ensembl (gene) annotation providing an RXE value of 0.34 while the RefSeq (protein coding) annotation gave a value of −0.17. Implementation of different genome annotations leads to contradictory conclusions; for example, in the 45, X^m^ lymphoblastoid cell line the X undergoes strong compensation with the Ensembl (gene) annotation, but undergoes incomplete dosage compensation with the RefSeq (protein coding) annotation (Table 3). In addition, we observe lower RXE values when comparing the RefSeq to RefSeq (protein coding) annotations, suggesting that non-coding transcripts play a significant role in dosage compensation. In our analysis, RefSeq likely provided the most consistent results due, at least in part, to the fact that its entries are non-redundant and are developed by evidence-based gene identification.

**Table 3:** RXE based on annotation

**Annotation type**	**Human liver**	**Human brain**	**Human lymphoblast 45, X**^**m**^	**Human lymphoblast 45, X**^**p**^
**RefSeq**	−0.28	0.16	−0.14	−0.19
**RefSeq protein coding**	−0.41	−0.11	−0.17	−0.25
**Ensemble (gene)**	−0.33	−0.09	0.34	0.02
**Ensemble (transcript)**	−0.12	0.09	0.01	−0.45
**UCSC (h19) known genes**	0.05	0.08	0.00	−0.66
**Gencode**	−0.07	0.09	−0.12	−0.38

Variation in relative X-chromosome expression values resulting from different genome annotations used in mapping. All data was log_2_-transformed. For each row, the same mapping file was used, but a different annotation was implemented in the program Cufflinks in order to generate FPKM values for each gene.

### Dosage compensation in tissues

**Figure 3:**
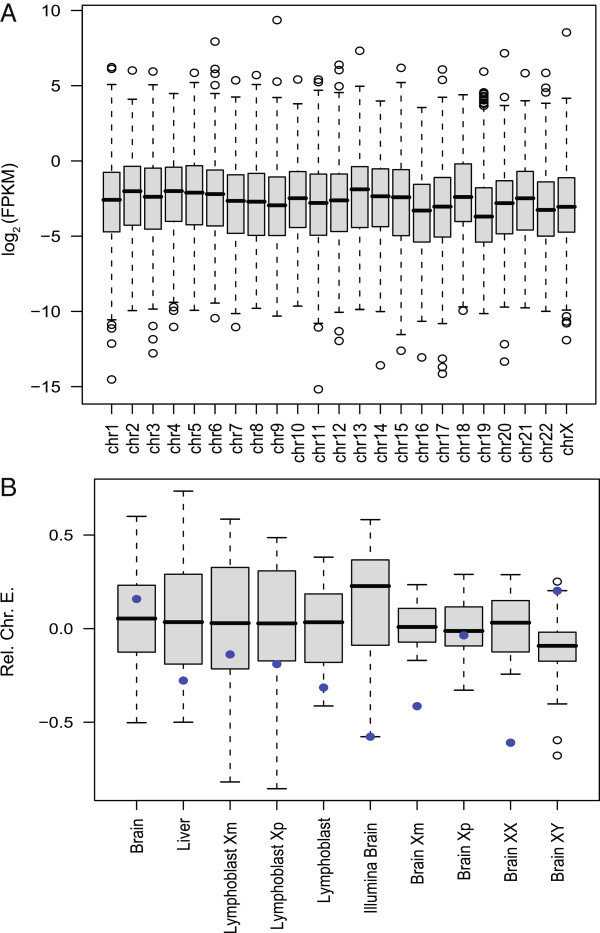
**Box plots of log**_**2**_**-transformed gene expression data.** (**A**) Boxplots of log_2_-transformed FPKM values for each chromosome in the human lymphoblast 45, X^p^ sample and (**B**) Boxplots of the average gene expression of each chromosome relative to the average gene expression of all autosomes. Blue dots indicate X chromosome values for each library. In B, note that a value of 0 indicates equal expression with other chromosomes, while a value of −1 indicates expression at half the level of other chromosomes.

Using transformed data with outliers removed and the preferred, aforementioned methods of “non-unique, spliced” mapping and RefSeq reference annotation implemented in Cufflinks, we determined that expression of X-linked genes exhibit a similar expression range when compared to that of any autosomal pair (Figure 3A, Additional file 5: Figure S1). Additionally, examination of the mean relative chromosomal expression value of any one chromosome compared to the average expression of all autosomal chromosomes (i.e. including A to A comparisons as well as X to A comparisons) and RXE showed that the RXE values fall well within the normal range for most samples (Figure 3B). The mean RXE values ranged from −0.61 to 0.20 in the 10 tissues; however, in only two tissues (mouse X^m^ brain and mouse XX brain) were those values less than expected from a typical distribution of any other chromosome-to-chromosome relative expression values. Alternative means of central tendancy summarization (i.e. median scores) also matched this pattern of partial to complete dosage compensation (RXE ranged from 0.047 to −0.58), but with slightly lower values. The mouse X^m^ brain and XX brain libraries represent the libraries sequenced with the lowest depth of coverage in our RNA-seq data collection, suggesting an interaction between sequence coverage and the analysis of global gene expression (discussed below). However, none of the RXE values for any tissue indicated a lack of dosage compensation (RXE = −1).

### Library preparation and depth of coverage

The final factors we found that heavily influence the calculation of global RXE are library preparation and size or depth of coverage. Of the 10 principal libraries used in this study, 6 were produced internally for the ABI SOLiD platform. The other datasets were obtained from public databases and were prepared for the Illumina deep sequencing platform. While protocols for library preparation for both platforms involve a step to remove rRNA from the sample before sequencing, the two differ in implementation. Library preparation for the SOLiD platform utilized ribo-depletion, a subtraction of ribosomal RNA using probes that specifically bind and remove rRNA, while Illumina library preparation generally used poly-A selection, which isolates mRNA from total RNA (and, thus rRNA). Poly-A selection enriches for processed mRNAs and is 3’-biased, while ribo-depletion does not exclude non-polyadenylated RNAs that may be non-coding. Analyzing the top 250 highest expressed genes, with no consideration to chromosomal location, we discovered that SOLiD libraries were typically enriched for small RNA genes (e.g. Sno and micro); whereas, Illumina libraries were enriched for riboprotein coding genes (Table 4, Additional file 6: Table S5). It is evident that these two transcript groups have a large influence on determination of FPKM values in their respective libraries given their inherent high rates of expression. However, mapping implemented in TopHat helped to mitigate the influence these different classes of transcripts have on genome-wide expression values.

**Table 4:** Small RNA and riboprotein enrichment based on library preparation (Illumina or SOLiD)

	**Tissue**	**Xp lymphoblast (SOLiD)**			**Human lymphoblast (Illumina)**			**XY mouse brain (SOLiD)**			**Mouse brain (Illumina)**		
	**Mapping parameter**	**Unique**	**Non-unique**	**NUS**	**Unique**	**Non-unique**	**NUS**	**Unique**	**Non-unique**	**NUS**	**Unique**	**Non-unique**	**NUS**
Category	Small RNAs	193	190	4	2	8	1	47	62	2	4	2	4
	Ribo	20	26	42	58	80	62	2	11	3	3	8	3
	Other	37	34	204	190	165	187	201	177	245	243	240	243

We compared small RNA (sno and micro) and riboprotein biases using three different mapping parameters: unique, non-unique, and non-unique splicing (NUS). Four different libraries were analyzed including: X^p^ lymphoblast (SOLiD), normal human lymphoblast (Illumina), mouse brain (Illumina) and mouse brain 40, XY (SOLiD).

To examine the effect of library size on RXE estimation, we included 9 more human lymphoblastoid cell line RNA-seq libraries from a recent study [31], for a total of 14 human RNA-seq libraries analyzed. We calculated the library size and the associated RXE value for each library (Additional file 7: Figure S2). As anticipated, the 10 human lymphoblastoid cell lines, which were relatively small libraries (<50 million reads), clustered together with RXE values ranging between −0.4 and −0.1. Smaller libraries also appeared to be more variable in their estimates of RXE. For the 45, X^m^ and 45, X^p^ lymphoblastoid cell line libraries, which were much larger (>250 million reads), the RXE values approached and surpassed 0. Overall, RXE values look to asymptote to ~1 as library size increases. Given this result and our observations about the divergent behavior of our smaller mouse libraries, it has been demonstrated that low coverage libraries lack the power to properly assess expression of lowly expressed genes and paralogs and, in turn, alter the final RXE values.

### Functional components of dosage compensation

**Figure 4:**
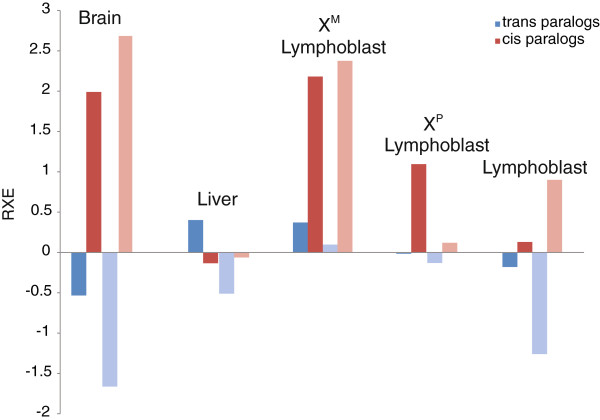
**Relative X-chromosome expression (RXE) values for *cis *and *trans *paralogs associated with GO terms binding activity (dark colors) and enzyme activity (light colors) for five human tissue samples.** RXE values were generated using non-unique, splicing mapping parameters. All samples, excluding liver, exhibited greater RXE values from *cis*-paralogs. Number of genes in each category on the X-chromosome and the average per autosome are listed in Additional file 8: Table S6.

**Figure 5:**
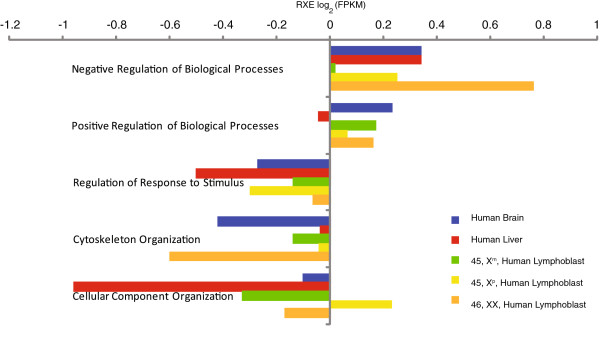
**RXE values for genes associated with specific Gene Ontology (GO) terms in five human tissues.** Number of genes in each category on the X-chromosome and the average number of genes per autosome are shown in the parentheses, respectively (N_X_, N_AVG. per__AA_). Sample sizes for the each group is as follows: Negative Regulation of Biological Processes: Brain – N_X_=66, N_avg. per AA_=99; Liver – N_X_=63, N_avg. per AA_=95; Lymphoblast X^m^ – N_X_=48, N_avg. per AA_=73; Lymphoblast X^p^ – N_X_=47, N_avg. per AA_=72; Lymphoblast XX – N_X_=64, N_avg. per AA_=98; Positive Regulation of Biological Processes: Brain – N_X_=86, N_avg. per AA_=108; Liver – N_X_=82, N_avg. per AA_=105; Lymphoblast X^m^ – N_X_=67, N_avg. per AA_=80; Lymphoblast X^p^ – N_X_=68, N_avg. per AA_=79; Lymphoblast XX – N_X_=88, N_avg. per AA_=108; Regulation of Response to Stimulus: Brain – N_X_=55, N_avg. per AA_=72; Liver – N_X_=52, N_avg. per AA_=69; Lymphoblast X^m^ – N_X_=36, N_avg. per AA_=51; Lymphoblast X^p^ – N_X_=38, N_avg. per AA_=50; Lymphoblast XX – N_X_=50, N_avg. per AA_=71; Cytoskeleton Organization: Brain – N_X_=21, N_avg. per AA_=25; Liver – N_X_=21, N_avg. per AA_=24; Lymphoblast X^m^ – N_X_=15, N_avg. per AA_=19; Lymphoblast X^p^ – N_X_=14, N_avg. per AA_=19; Lymphoblast XX – N_X_=22, N_avg. per AA_=24; Cellular Component Organization: Brain – N_X_=98, N_avg. per AA_=111; Liver – N_X_=95, N_avg. per AA_=105; Lymphoblast X^m^ – N_X_=75, N_avg. per AA_=76; Lymphoblast X^p^ – N_X_=70, N_avg. per AA_=84; Lymphoblast XX – N_X_=101, N_avg. per AA_=108.

Selection for dosage compensation of a particular gene will depend on its stoichiometric relationship to its functional partners. In order to see if gene function corresponds to a tendency for compensation we examined RXE for *trans-*paralogs only and *cis-*paralogs only in relation to GO-term Molecular Function categories previously identified to be susceptible to gene dosage effects [35]. In the GO-categories Binding Activity and Enzyme Activity, all tissues except for liver showed higher RXE values for *cis-*paralogs than *trans-*paralogs (Figure 4). The “gene balance hypothesis” [36] suggests that dosage compensation would be more likely to be found in gene networks that require many components for proper functionality and are, thus, dosage-sensitive. Regulatory processes are identified as likely candidates for this type of constraint. For GO-term Biological Processes categorical groups, both Negative Regulation of Biological Processes and Positive Regulation of Biological Processes had RXE values indicative of being dosage compensated. Other groups, whether they were possibly related to regulatory process (e.g. Regulation to Response to Stimulus) or core cellular processes (Cytoskeleton Organization and Cellular Component Organization), showed less evidence for strong selective pressures for dosage compensation (Figure 5). These results are essentially in agreement with the recent report by [37].

## Discussion

Given the variable conclusions reached in several investigations concerning sex chromosome dosage compensation in different organisms [38-42], how confident can we be that any particular report has accurately measured expression levels clustered by a chromosome-to- chromosome level? Recently, data from a previously reported non-dosage compensated Z-chromosome in the silkworm [39] has now been re-analyzed with consideration for statistical biases and concludes that the Z is being dosage compensated, rejecting the premise that ZW sex determination necessitates deviation from dosage compensation [43]. Our analysis of RXE in human and mouse identified similarly serious issues that are not only important to the specific question of dosage compensation but address broader issues concerning the implementation of analytical tools for next-generation sequencing data. While our analysis focused on chromosome level comparisons, the issues we address will likely impinge on conclusions drawn from many types of global or clustered analysis of short read sequences. Our examination of the effects of library construction/sequencing methods, mapping protocols, sequence annotations and statistical treatment of data on estimates of RXE may also prove to be incomplete as RNA-seq data analysis continues to mature. The pitfalls we illustrate for RNA-seq are similarly presented for repetitive elements in short read genome assemblies by [44].

Three key questions considered in mapping short read sequence to a reference genome have a profound effect on downstream quantitative analysis of RNA-seq datasets: 1) are reads that align to more than one location in the reference reported in the mapped dataset; 2) if so, how many of those alignments are reported; and 3) if reporting of multiply-aligning short reads is limited, what rules govern the location to which a reported short read is assigned? Unique mapping parameters, implemented in a mapping program such as Bowtie, typically elide any reads that align to more than one location, hence genes that contain even short segments of high similarity to other genes will be excluded from further analysis. Depending on the limits to reporting of multiply-aligned read, “non-unique” parameters, may either swamp quantitative analysis with inclusion of high copy-number repeat transcripts or lead to inappropriate inclusion of non-expressed paralogs. Default parameters in programs such as TopHat and Cufflinks report multiply-aligned reads that may dramatically influence conclusions drawn in clustered analyses. Our analysis shows that the X chromosome is enriched for paralogous gene families relative to the autosomes. Since gene duplication is a straightforward method for achieving dosage compensation of a haploinsufficient gene, implementation of short-read sequence analysis tools that are inclusive of limited multiply-aligned sequences is essential to generating the most biologically realistic RXE levels.

Another consideration that appears to have a significant effect on the calculation of RXE is the use of a mapping tool that includes splice junction fragments. Consideration of splice junction fragments removed biases created by enrichment for small RNAs or riboproteins that were introduced during library preparation for either SOLiD or Illumina platforms. Differences in the consideration of splice junction fragments may also underlie the large discrepancy in RXE values produced from using different gene annotations. While all tissues across all annotations exhibited higher levels of RXE than those described in [19], we found considerable variation in RXE estimates when comparing values between all five annotations. This is of particular concern considering that some of these comparisons should be very similar. For instance, Ensembl uses Gencode annotations in the formulation of Ensembl genes and Ensembl Transcripts annotations. The fact that choice of annotation for mapping assignment is an unexpectedly important facet of RNA-seq analysis has also been reported by others [45].

Previous RNA-seq studies of X:autosome expression applied arbitrary cutoffs when filtering data, removing a proportion of genes that are highly or lowly expressed [19-24]. In each of these reports the approach to data trimming can be seen to improve the fit of the calculated X:autosome expression ratio to the authors’ desired conclusion. Trimming according to expression level clearly introduces bias because compensated genes may be disproportionately represented within different expression level classes. In other words, excluding genes with low, or high, FPKM values may result in exclusion of a significant cohort of compensated X-linked genes. Most FPKM estimation programs such as Cufflinks have some type of threshold criteria for determining whether or not a FPKM value will be called for that locus. Ascertainment bias from arbitrary cutoffs will be particularly acute for smaller libraries. Many RNA-seq studies, including the X dosage reports discussed herein, cite [29] for the designation: 1 transcript per cell is equivalent to FPKM=3. It should be noted that that equivalency only holds for the specific approach (i.e. RNA source, library preparation, mapping parameters) used in [29]. This is particularly the case when using Cufflinks, in which FPKM estimates, without some sort of standard reference, are meaningful only in the relative sense. Recent studies indicate biologically relevant transcripts are represented at much greater depth [46,47] and need to be accounted for in mapping and transcript assembly.

We found library size and type are very important in interpreting global expression analysis. The decision about which method of library construction to use can have a profound influence on characterization of expression profiles [48]. In our study we included results from both Illumina RNA-seq and SOLiD RNA-seq, revealing differences between the two platforms largely due to the method of rRNA exclusion in library construction. Our comparisons of non-coding versus protein-coding annotations show that methods that exclude non-coding elements present lower estimates of RXE. Sequencing depth also plays a role in accurately modeling global or clustered gene expression. Library size and RXE are positively correlated in our analyses. Recent studies have indicated that a lack of sequencing depth is typically associated with the inability to detect lowly expressed genes [46,49-51].

The evolution of dosage compensation of sex-linked genes will be driven by the fitness cost of under-expression in the heterogametic sex weighed against the cost of over-expression in the homogametic sex on a gene-by-gene basis. It is expected that only some genes will necessitate compensation once they become hemizygous. Therefore, gene function and the relative representation of certain functional groups on the sex chromosomes becomes an important consideration in the detection of dosage compensation at the chromosome level. Although only weakly supported, our RXE calculations with respect to gene ontology classification largely agree with the predictions of the gene balance hypothesis [36], in which regulatory genes tend to be compensated while structural genes tend not to be.

High-throughput gene expression profiling forms the experimental basis of several recent reports that show either no evidence for dosage compensation such as in birds [8,42] and lepidoptera [39], or that show some amount of dosage compensation such as in platypus [38], stickleback [40], and flour beetle [41]. Even with the greater sensitivity afforded by next-generation sequencing and RNA-seq analysis, the choice of analytical tools and decisions implicit in their implementation, particularly with respect to inclusiveness of data, will have a profound effect on the conclusions drawn in any clustered analysis. More importantly, as others have argued, compensation may be more local than global [11,36]. In the absence of an overriding chromosome-wide epigenetic mechanism, detection of dosage compensation for a sex chromosome will clearly depend mostly on the relative number of dosage sensitive genes to dosage insensitive genes that reside on it.

## Conclusions

Our analysis of RNA-seq data, in consideration of several mitigating factors, indicates that gene expression from the X chromosome in mammals is up-regulated in many somatic tissues. While not every tissue-specific RNA-seq dataset has an RXE ≥ 0, no tissue in our analysis exhibits RXE as low as the values reported in [19]. Some of these differences in RXE can be attributed to tissue specific activity of X-linked genes [52], however we find RXE values falling within the range of variability of other chromosome-to-chromosome expression ratios. In addition, we identified serious issues not only important to addressing dosage compensation but to the larger concern of accurately implementing analytical tools for next generation sequencing. Our study shows how choices made along the entire pipeline of next-gen sequence analysis can profoundly influence the final conclusions to questions asked by many biologists.

## Methods

In order to generate an global estimate for the relative expression of the X-chromosome to the autosomes, we implemented an analytical framework to RNA-seq that involved taking into consideration the methods and various assumptions associated with each methodological step: library construction; sequencing run; mapping reads from sequencing runs to a reference genome; assigning those mapped reads to annotated region of interest; calculating an expression value for that region of interested largely based on the mapping of those reads.

### Mapping

We implemented three different mapping protocols in our study to address three specific base assumptions about how mapping should be done and are referred as follows: “unique”, “non-unique”, and “non-unique, spliced”. Mapping runs were conducted using the Bowtie v0.12.7 algorithm and program [53]. “Unique” means that a read is only included in the mapping results file if it maps to only one unique location in the reference. In terms of Bowtie parameters, this means that parameter k and m were set to 1. If the read maps to more than one region of a reference, then it is discarded from downstream expression estimates. A “non-unique” approach allows those reads that map to multiple locations in the reference to be included in downstream analyses. To isolate the effect of simply including multiply-mapped reads, our “non-unique” mapping allows for multiply-mapped reads to be report but only once. For this approach, Bowtie parameter k was set to 1, while m had no limit. Additionally, all subsequent mapping matches for a read were ranked using the “best” and “strata” algorithms within Bowtie that rank the matches for a specific read using the number of mismatches within seed and across the entire read as well as the Phred scores at those mismatches. Our “non-unique” analysis only reports the “best” ranked match for a mapped read. Lastly, a “non-unique, spliced” mapping approach is most commonly recognized and implemented in the TopHat v1.3.1 program [33], which includes the consideration of splice junctions for discontinuous mapping of reads. All default parameters were used for these runs; however, no novel transcripts were predicted as Gencode v4 and mm9 USCS gene models were used to define all splice junctions for human and mouse, respectively. This approach allows for non-unique mapping as well and uses a similar methods of assigning alignment scores, but reports up to 20 randomly selected sequences if alignment scores are identical (default setting). To detail whether the distribution of genetic entities such as paralogous genes among chromosomes might bias a specific mapping strategy, paralogs were identified using BioMart and differences in read mapping for those paralogs with >70% sequence similiarity were examined for both unique and non-unique mapping runs. Our 70% minimum cutoff for paralogs was empirically determined by the ability of the Biomart paralogs search algorithm to identify X-linked multigene families (eg. *Xlr*) of which we had prior knowledge.

### Reference annotation

To describe the role that reference annotation had on RXE, we examined different approaches to assigning reads for expression calculations: (1) mapped reads only, disregarding a priori regions of interest (such as exonic regions); (2) RefSeq exon annotations for genes to determine which reads mapping to specific regions we would be retained in our estimates of expression; (3) RefSeq exon annotations for protein coding genes only; (4) Ensembl exon annotations for genes; (5) Ensembl exon annotation for transcripts; and (6) Gencode exon annotations. For approach (1), we estimated relative expression by weighting the number of reads that mapped to any specific chromosome by the number of genes found on that respective chromosome (patterns of chromosomal gene-enriched were described using BioMart) and then dividing the weighted number for the X-chromosome by that number. By averaging values of this relationship across all chromosomes, we calculated a value for RXE for each library that we examined (data was log2-transformed to maintain consistency for reasons described below). Alternatively, for approaches (2)-(6), we implemented the program Cufflinks v1.0.3 [34] to estimate fragments per kilobase of exon per million fragments (FPKM) – an index typically used in RNA-seq analyses – using different annotations with the same mapping results files. Default parameters were used for all Cufflinks FPKM calculations except for limiting FPKM calculations to the sites determined by the aforementioned associated annotations (without allowing additional transcript prediction). All multi-mapped reads contributions to FPKM values are equally distributed across all valid mapping sites (i.e. if a single read maps to 10 sites, then each sites is awards 1/10^th^ of that read to its total read count). Software-based bias corrections (both Fragment and Multi-map) were implemented but neither had any significant effect on results.

### Data manipulation and selection

We implemented three treatments of raw results to increase impartial statistical rigor, the amount of data used in the analysis, and overall robustness of analysis: (1) we log-base two transformed all FPKM values; (2) we removed any outliers that were 1.5 times the mid-50 percentile distance greater or less than the 75th and 25th percentiles, respectively; and (3) we used mean values and instead of median values. A log_2_-transformations of data changes the scale of analyses and allows for more appropriate assessment of lowly-expressed loci (particularly, FPKM values <1) and highly-expressed loci (reducing effects of large values on moment estimation) by allowing the distribution of data to closely resemble a “normal” distribution model and, thus, better describe the central tendencies of that distribution. Taking an impartial approach to outlier identification minimizes differences among tissues with very specific patterns of gene expression and removes data points that may overly influence mean estimation of a general pattern in relative X and autosomal gene expression while maintaining statistical rigor.

Given that we used a log_2_-transformation, instead of the traditional X:A ratio (described as X expression divided by autosome average expression), we used an index of log_2_(X expression) – log_2_(A expression) to describe patterns in relative X expression, or RXE. Here, a value of zero means equal expression of X and A, a value of 1 means twice as much expression of X than A, and a value of −1 means half as much expression on the X as compared to A. Therefore, it follows that values near zero indicate dosage compensation, while values near −1 indicate no dosage compensation occurring.

### Final relative X-chromosome expression estimation

Using information gathered from the above treatments of data, we decided on using a “non-unique, spliced” approach to mapping that uses the conservative gene-identifying RefSeq annotation and log_2_-transformation of FPKM values with a traditional approach to outlier removal to estimate RXE. In addition to estimating RXE, we also calculated the relative expression of each chromosome to all other chromosomes (excluding the Y and mitochondria) in order to see if the X truly deviates from the expression patterns of other chromosome (i.e. is at half the expression level of other chromosomes).

### Functional component dosage compensation

All FPKM values for 5 human tissue-types (brain, liver, lymphocyte, lymphocyte X^M^, and lymphocyte X^P^) were filtered based on the GO-term of interest by mining the on-line AMIGO database for gene names associated with each Biological Process term of interest. Molecular Function group comparisons were done in a similar fashion, however, the identification of term of interest was based on results by Kondrashov and Koonin’s [35] that found some specific terms to be overly-represented in haplo-insufficient genes.

### Data access

GSE16921; GSE12946; SRA001030; SRA047980

## Abbreviations

RXE: Relative X expression; XCI: X chromosome inactivation; FPKM: Fragments per kilobase of exon per million fragments mapped; Xm: Maternal X chromosome; Xp: Paternal X chromosome; NUS: Non-unique, spliced; GO: Gene ontology.

## Competing interests

The authors declare that they have no competing interests.

## Authors’ contributions

NJ and MM designed and performed RNA-seq, assembled and analyzed all data sets. SK, SQ, CO participated in design and performance of RNA-seq and analysis, SE and RF participated in experimental design and data analysis. RO and MO conceived and designed the study. NJ, MM, SK and MO wrote the manuscript. All authors read and approved the manuscript.

## Supplementary Material

Additional file 1: Table S1 Proportion of total genes removed through implementation of methods from cited dosage compensation studies. Proportion of total genes that yielded a FPKM value > 0 that would not be included in the final calculations of RXE as defined by of the authors in the methods of the studies referenced below. ^a^ Utilizing described Miller’s Jackknife/Mann–Whitney U-test approach, applied unique mapping, established all genes that had a FPKM of zero, then removed a compensatory amount of genes from the upper end of the distribution. ^b^ Applied unique mapping, then removed all genes that had a FPKM <1. ^c^ Applied non-unique, splicing mapping, then removed all genes that had a FPKM <1. ^d^ Applied non-unique, splicing mapping, then removed outliers. Click here for file

Additional file 2: Table S2 Paralog enrichment by chromosomal location in both mouse and human. Paralogous transcripts were determined by using BioMart (Ensembl), isolating paralogs with identity >70%. Number of genes per chromosome was calculated using RefSeq genome annotation. Click here for file

Additional file 3: Table S3 RXE comparison using analysis methods implemented in cited dosage compensation studies. ^a^ Applied unique mapping. included all expressed genes and reported median values. ^b^ Applied unique mapping, then removed all genes that had a FPKM <1 and reported median values. ^c^ Applied non-unique, splicing mapping, then removed all genes that had a FPKM <1. Gene expression was calculated using cufflinks algorithm for all three analysis strategies to produce RXE values. Click here for file

Additional file 4: Table S4 Reads and genes mapped by mapping parameter, for libraries analyzed in both mouse and human. Click here for file

Additional file 5: Figure S1 Box plots of log_2_-transformed data of all FPKM values by chromosomal location. (A) human brain, (B) human liver, (C) normal human lymphoblast, (D) X^m^ human lymphoblast, (E) X^m^ mouse brain, (F) X^p^ mouse brain, (G) 40, XX mouse brain, (H) 40, XY mouse brain, (I) mouse brain. Click here for file

Additional file 6: Table S5 Small RNA and riboprotein enrichment based on library preparation (Illumina or SOLiD). We compared small RNA (sno and micro) and riboprotein biases using three different mapping parameters: unique, non-unique, and non-unique splicing (NUS). Six different libraries were analyzed including: X^m^ lymphoblast (SOLiD), human brain (Illumina), human liver (Illumina) and X^m^ mouse brain (SOLiD), X^p^ mouse brain (SOLiD), and 40, XX mouse brain (SOLiD). Click here for file

Additional file 7: Figure S2 Library size affects relative X-chromosome expression values in mammalian tissues. Plot of average log_2_-transformed RXE based on number of reads mapped. Includes data from human lymphoblast 45, X^m^ (n=1), human lymphoblast 45, X^p^ (n=1), human lymphoblast (n=10), human brain (n=1), and human liver (n=1) RNA-seq samples. Click here for file

Additional file 8: Table S6 Gene counts for relative X-chromosome expression (RXE) values for *cis* and *trans* paralogs associated with GO terms binding activity and enzyme activity for five human tissue samples as described in Figure 4. Click here for file
